# Qualitative study on custodianship of human biological material and data stored in biobanks

**DOI:** 10.1186/s12910-016-0098-0

**Published:** 2016-03-01

**Authors:** Michiel Verlinden, Herman Nys, Nadine Ectors, Isabelle Huys

**Affiliations:** Clinical Pharmacology and Pharmacotherapy, KU Leuven, Leuven, Belgium; Interfaculty Centre for Biomedical Ethics and Law, KU Leuven, Leuven, Belgium; AC Biobanking, UZ Leuven, Leuven, Belgium; Translational Cell & Tissue Research, KU Leuven, Leuven, Belgium; Centre for Intellectual Property Rights, KU Leuven, Leuven, Belgium

## Abstract

**Background:**

Balancing the rights and obligations of custodians and applicants in relation to access to biobanks is of utmost importance to guarantee trust and confidence. This study aimed to reveal which issues divide different stakeholders in an attempt to determine the rights and/or obligations held on human biological materials (HBM) and data.

**Methods:**

Twenty-eight informants in the Benelux and Scandinavia were interviewed in order to capture the perspectives of experts and stakeholders in relation to the rights and obligations held by custodians and applicants with respect to access to HBM and data.

**Results:**

There was no consensus among the informants on whether the custodian of a biobank should decide upon the scientific merits and the utility of an access request. Nearly all informants agreed that a new request or an amendment to the initial request has to be submitted when an applicant wants to use leftover HBM in a new or follow-up project. Several informants felt that it might be justified to charge higher access fees to external or industrial applicants that did not contribute (directly or indirectly) to the collection of HBM and data. Most informants agreed that a custodian of a biobank could request the sharing and return of research results. It was furthermore argued that some of the benefits of research projects should be fed back into biobanks.

**Conclusions:**

The interviews revealed a rather complex web of rights and obligations allocated to the custodian and the applicant in relation to access to HBM and data stored in biobanks. Some rights and obligations are negotiated on a case-by-case basis, while others are stipulated in access arrangements. We did find a consensus on the attribution of certain general rights to the custodians and the applicant.

**Electronic supplementary material:**

The online version of this article (doi:10.1186/s12910-016-0098-0) contains supplementary material, which is available to authorized users.

## Background

Researchers need to be able to access efficiently different collections of HBM and data [1, 2]. However, biobanks and biobank networks, as custodians of HBMs and associated data, need to exercise a certain control on the access to and the use of their collections in order to guarantee their long-time sustainability and the scientific, ethical and legal correctness of their use [2–5]. In addition, custodians need to ensure that access requests comply with the applicable legislation and the conditions stipulated in the consent of the donors/patients [6, 7]. Balancing the rights and obligations of custodians and applicants in relation to access to biobanks is of the utmost importance to guarantee trust and confidence.

After many years of discussions there is still no answer to the question whether ‘ownership’ rights can be held or claimed in relation to HBMs (and associated data) collected and used for research purposes [2, 8–12]. Considering the uncertain status of the ‘ownership’ of HBM, some authors suggested applying instead the concept of ‘custodianship’ [13], ‘charitable trust’ [14] or ‘stewardship’ [15]. However, replacing the concept of ownership with new concepts does not automatically provide an answer to the practical question as to which rights and obligations can be held on HBM and data, as rightfully indicated by J. Conley e.a. [16]: *“When the National Cancer Institute announces that (…) it will rely on the poorly defined state-law concept of “custodianship,” it seems less a solution than an invitation to even greater inefficiency. The same applies to Winickoff and Winickoff’s “charitable trust” analogy and the “stewardship” proposal advanced by Fullerton et al.: because of the legal imprecision of their central concepts and the apparent complexity of adapting them to biobanking, they are, on a practical level, likely to introduce more problems than they solve”* [16].

Previous empirical research on biobanks focused on consent, public perception and participation in biobanks, etc. [17–22]. Few research projects have focused on custodianship of HBM stored in biobanks [23–25].

The qualitative study described in this paper, has looked into the following research questions:

- What are the different perspectives held by stakeholders in relation to the (bundle of) rights and obligations held by custodians and researchers applying for access to HBM and data?
- Which topics divide the different stakeholders in trying to determine the (bundle of) rights and obligations held by custodians and applicants?

## Methods

### Definitions

In this article the term ‘access arrangements’ can be defined as ‘*guidelines, best practices, opinions, policies, agreements, etc. containing rules on access to and use of HBM and data collections stored within biobanks*’ (a similar definition of access arrangements is used by S. Fortin et al. [4] and the OECD [26]). An ‘access committee’ can be defined as ‘a committee established by a biobank to decide upon a request for access to the collection of HBM and/or data stored in the biobank.’ The ‘biobank’ refers to ‘a (single) infrastructure dedicated to the storage and provision of HBMs or data or both for research purposes.’ The term ‘biobank network’ refers to ‘a group of institutions that freely assume the commitment to collaborate in the domain of biobanking and that (often) share the same procedures and quality policies, and that are (or might be) helped by a central hub for coordination in terms of service’ [5]. ‘Custodianship’ can be defined as the ‘caretaking responsibility for HBM and data that starts at the planning of a biobank initiative, prior to the collection, and continues through research use to final dissemination of research results’ (a slightly adapted version of the definition used by R. Yassin et al.[13] and the National Cancer Institute [27]). The ‘custodian’ is defined – by the authors – as the person(s) or entity – such as the biobank manager – and/or the access committee – and/or the institution that exercises custodianship on HBM and data stored in a particular biobank. The term ‘ownership’ can be defined as ‘the ultimate and exclusive right conferred by a lawful claim or title, and subject to certain restrictions to enjoy, occupy, possess, rent, sell, use, give away, or even destroy an item of property’ [28]. Finally, the term ‘researcher’ refers to researchers both from the public and private sector.

### Data collection: key informant interviews

Data were collected via key informant, face-to-face (and some Skype-based) interviews. All informants confirmed their participation in an email. The study did not require an ethical approval, since it is non-experimental and we did not interview human research subjects (i.e., patients or donors) or health care providers. We furthermore did not obtain identifiable private/personal information from research subjects. This was confirmed by an analysis of the applicable regulations in the concerned countries.

At the beginning of the interview the informants were asked whether they objected to the fact that the interviews would be recorded and transcribed *ad verbatim*. None of the informants made any objection and all of them answered our questions voluntarily which confirms their consent to participate.

The aim of the interviews was to gain an in-depth understanding of how access to data is arranged in the daily practice of biobanks and biobank networks. A qualitative research method was chosen to gather information on the hopes and concerns of the different stakeholders in this respect. Semi-structured interviews were conducted to collect information about a number of pre-defined topics (enlisted in the interview guide (Additional file 1), at the same time allowing the interviewers to probe deeper when required. The interview questions deliberately did not explicitly refer to legal concepts such as rights and obligations, in order to avoid that the informants would focus their answer on such concepts.

### Sampling a strategy for the key informant interviews

**Fig. 1:**
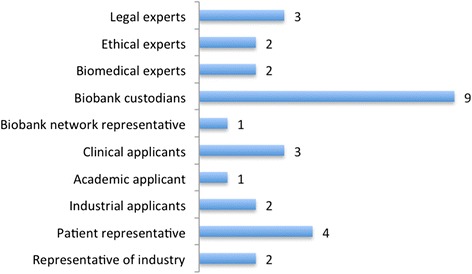
Distribution of informants per type of stakeholder or expert

Twenty-eight informants in Europe were selected by means of ‘purposeful/purposive’ sampling [29, 30] and snowball sampling based on the information provided by previous informants. The purpose of the sampling was to capture the different perspectives of 4 distinct categories of stakeholders – in particular (i) custodians of biobanks and custodians/representatives of biobank networks, (ii) clinical, academic and industrial (research) applicants, (iii) patient representatives and (iv) representatives from the pharmaceutical industry. In case informants fulfilled a double role, the interviewers considered those double roles focusing on the main role of the informant. We also conducted interviews with legal, ethical and biomedical experts (see Fig. 1). The interviews with the 4 categories of stakeholders enabled us to acquire a deeper knowledge of the current practices applied in biobanks and biobank networks and HBM research in general. Interviewing legal, ethical and biomedical experts provided a more in-depth understanding of the (ethical, legal and scientific) background and context in which biobanks operate.

The authors wanted to study variations in perspectives between different categories of stakeholders and experts in relation to the rights and obligations held by the custodian and the researcher/applicant. That is why it was attempted to select at least two informants to represent each category of stakeholders and experts. The analysis of the interviews exposed fewer variations in opinions than expected. It was also noticed that informants representing the same categories of stakeholders or experts expressed as much variation in their opinions as informants who belonged to different categories of stakeholders or experts. This limited variety in opinions might be attributed to the fact that some of the topics discussed during the interview were relatively new for some informants. For instance, some informants explicitly mentioned that they did not yet have a fully developed opinion on the right to share benefits. It would be interesting to conduct more interviews and possibly discover a larger variety of opinions. Unfortunately, due to time constraints it was not possible to interview additional informants at this stage.

### Geographical scope of the interviews

**Fig. 2:**
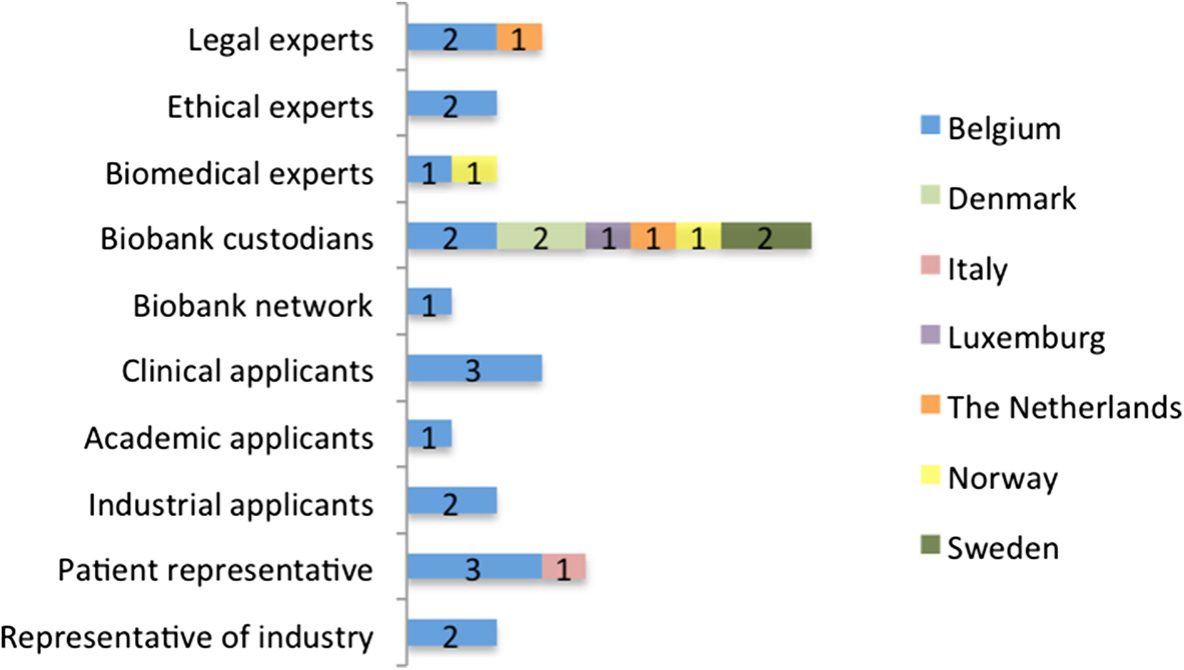
Geographical distribution of stakeholders and experts. One informant was active in two different countries

Between October 2013 and January 2014 21 informants were interviewed in the Benelux. Those countries were chosen since they host ambitious national biobank initiatives such as the Belgian Virtual Tumour Bank and the Centre for Medical Innovation in Belgium, the Parelsnoer Institute, BBMRI.NL and LifeLines in the Netherlands and the Integrated BioBank of Luxemburg (IBBL). In February and March 2014 interviews were conducted with five informants in Denmark, Sweden and Norway (see Fig. 2). The Scandinavian countries are considered pioneers in the development of biobanks and biobank networks and in epidemiological research [31] since they have a long tradition of storing HBM and health data of patients within the framework of health care services and population-based studies. An interesting dimension of the Swedish biobank landscape is the well-documented cooperation between university biobanks and pharmaceutical companies. Additionally, a representative of an Italian patient organization was interviewed, since this organization represented patients with a rare disease. We combined the interviews with on-site visits of biobanks in Belgium, Denmark and Sweden to observe their existing policy and practices in relation to access to biobanks.

### Data analysis of the interviews

The data analysis phase consisted of an inductive analysis according to the Qualitative Analysis Guide of Leuven (QUAGOL) [32]. First, the transcriptions of the interviews were thoroughly reread by two members of the research team. Second, a narrative interview report was drafted to articulate the essence of the interviewee’s story in answer to the research questions. In a third stage, conceptual interview schemes were created. In the fourth stage, the interviews were reread again to verify whether the content of the conceptual interview schemes reflected the most important concepts aimed at in the research questions. The fifth stage consisted of a comparison of the conceptual interview schemes of the different interviews to identify and adjust common themes, concepts or hypotheses.

The actual coding process started with compiling a list of common concepts (stage six) (see Additional file 2). The resulting list of concepts was introduced as preliminary codes in the software program ‘Dedoose’. In the seventh stage, each significant passage of the interview was linked to one of the concepts of the list. In stage eight, every concept was analysed through a careful exploration and study of all citations associated with each concept. The ninth stage consisted of the extraction of the essential structure of all the interview data. Based on this conceptual framework and the in-depth analysis of stage 8, the authors were able to describe essential findings in answer to the research questions. Significant quotes were added where necessary and relevant. Finally, a formal peer debriefing with the other members of the research team was conducted to discuss and check the results in answer to the research questions.

## Results and discussion

### Which committee or body should decide upon access requests?

The legal framework of many countries – including the countries that were the object of this study – stipulates that Research Ethics Committees (RECs) need to provide an independent opinion on studies on human beings. More recently, RECs were also given the competence to provide an opinion on research projects that use HBM and data stored in biobanks. In some countries RECs are not expected to review the scientific quality of applications, while in other countries they are expected to make such an evaluation.

A previous empirical study [33] revealed that the majority of biobanks establish their own committees to exercise a certain control on access to their collections of HBM and data. Such access committees are established by a biobank independently from RECs and consist of different stakeholders involved in the functioning of the biobank. Authors have stressed the importance of clearly defining the mandate of access committees and the criteria and procedures used to decide upon an access request [4, 24, 34]. One will notice in this respect that article 14 (c) of Recommendation 2006 (4) provides that one should specify the conditions governing the access to and the use of HBM. Several authors are in favour of giving the access committee a rather broad mandate to decide upon access requests, including the scientific quality of the research project [35–37]. However, one author argued that only the biobank – represented by the custodian – has the right to finally decide on access request [38].

All interviewed informants agreed that access committees could decide upon the availability and suitability of HBM and data for particular research projects and the impact of the provision of HBM and data on the existing collection.

However, there was no consensus on whether custodians should decide upon the quality, the usefulness and the ethical value of research projects. Some informants were in favour of the idea that the custodian would follow the advice of a REC or funding body in this respect.

A custodian of a biobank in the Benelux stated the following in this respect:

“*The medical ethics committee is in my opinion not capable of evaluating the scientific part. It can evaluate whether something is ethically acceptable; that is why they are there, not to evaluate the scientific content. (*Free translation by the authors of a excerpt in Dutch*)*.”

The majority of the informants felt that the custodian – represented by an access committee – could decide upon the quality and/or scientific, societal and medical usefulness of the research project that requests access to the collection of HBM and data. As to the aspects assessed in such evaluation, these could include whether the suggested project (i) contributes to the improvement and/or accessibility of health care; (ii) corresponds to a high medical need; (iii) adds value to the existing state of science; and (iv) might lead to significant new (scientific) insights or outcome.

A representative of industrial applicants in the Benelux stated the following in this respect:

“*Nobody can predict the future to foresee whether we will receive within 6 months … a research application that is more relevant in relation to the (useful) knowledge that it will provide us. (…). So, I think it constitutes an important responsibility and a difficult exercise. (…) If all evaluation criteria are positive, I feel that one should move ahead (with the project) (*Free translation by the authors of a excerpt in Dutch*)*”

Some informants stressed the importance of clarifying and stimulating the interaction between access committees and RECs (and possibly also funding bodies) in the evaluation of an access request [34]. One possibility would be that the activities of access committees would be supervised by RECs [10]. The future article 22 § 1 of the Belgian Act on HBM provides that the aims and the activities of a biobank have to be reviewed by a REC.

Several informants stressed that access committees and/or RECs should avail themselves of the necessary expertise and experience to decide on access requests. An access committee should comprise representatives from different medical disciplines (such as pathology and surgery) as well as clinicians that collected HBM and data for the biobank, patient representatives and legal, ethical and biomedical experts. Another important requirement advanced by the informants is the need for profound guarantees that the committee or body that decides on access requests can act sufficiently independently from the researcher/principal investigator who requests access to HBM and data from the biobank. Such independence could be ascertained by involving one or more external experts in the evaluation of access requests. One should, of course, make sure that such an external expert does not have a conflict of interest in the evaluation of a particular access request. Several authors shared the opinion that access requests should be decided upon by independent committees that have the disposal of the necessary multidisciplinary expertise [10, 34, 35]. This requirement was motivated by the desire to avoid potential conflicts [13], to protect the interests, safety and wellbeing of the donors and to ensure that HBM and data are used in a meaningful way [35]. Article 19 of the Recommendation 2006 (4) of the Council of Europe [39] contains the requirement to establish an independent oversight of population biobanks, as well as regular audits of the implementation of the procedures that apply to access to and use HBM. Article 24 of the Recommendation 2006 (4) stipulates the obligation to conduct an independent examination of the scientific merit of a research project, the importance of the aim of research and the verification of the ethical acceptability. It should be pointed out, however, that the current text of Recommendation 2006 (4) only contains rules on RECs and does not relate to access committees.

Two informants from the Benelux with a legal background suggested that an equitable and proportionate set of access criteria could be determined via binding (national or European) legislation and that the custodian should only be granted a limited discretionary power to apply such criteria. They also suggested establishing some kind of appeal body that could verify whether the decisions of an access committee apply the access criteria in a non-discriminatory, objective and transparent manner.

Finally, access committees could be expected to sufficiently motivate their decision and policy.

All informants agreed that no different evaluation criteria should apply for academic and non-academic applicants and for external applicants and applicants affiliated to the biobank.

### Limited right of the custodian to decide on access to HBM and data

The different conditions that a custodian has to fulfil in the evaluation of access requests imply that the custodian is not entirely free to decide how to allow access to his collection of HBM and data. This could be justified by the fact that the custodians need to guarantee the protection of the rights and interests of the donor and/or the applicant. It raises the question whether an applicant could claim a fair or equal right to access and use of (publicly funded) collections of HBM and data. If such a right would exist, it would in any case not be absolute. The applicant can only use HBM and data for a certain period of time and for a research project that corresponds with the informed consent and the approval by a REC. It is therefore no surprise that the informants supported the idea that the custodian could require applicants to provide a short synopsis of the research project. There was no consensus on the extent to which applicants could be required to submit a more elaborate protocol with a description of the objective(s), design, methodology, statistical considerations and organization of a project. Such a requirement is often applied in the context of clinical studies on human beings and was suggested in a report of the National Cancer Research Institute, the National Cancer Intelligence Network and onCorde UK [37].

### The right of the custodian to decide on the fate of leftover HBM at the end of a project

Table 1 mentions the most important criteria suggested by informants to decide on the fate of leftover HBM. Nearly all informants agreed that the applicant should, at least, inform the custodian when HBM is leftover at the end of a project. There was further consensus on the fact that an applicant that wants to use leftover HBM in a new or follow-up project, has to submit a new request or an amendment to the initial request. The access committee or REC will need to approve such a request or amendment. All informants also agreed on the fact that the custodian could prohibit the applicant from transferring HBM (and data) to a third party (see Table 2). Taking into account the above, it may be concluded that the informants recognize the right of the custodian to decide – whether or not in collaboration with the REC – upon the fate of leftover HBM.

**Table 1:** Overview of criteria suggested by informants to decide on leftover HBM

Type of HBM	
Rare or common HBM	
Amount of leftover HBM	
Quality and reusability of leftover HBM	
Previous experiences and/or collaborations	
Cost and complexity of return

**Table 2:** Level of consensus on fate of leftover HBM

Return or destruction	No consensus
Re-use	Approval of access committee (and REC)
Transfer to third party	Approval of access committee (and REC)

There was no consensus on the criteria that should determine whether the biobank should require the return or destruction of leftover HBM. This may not be surprising, since other studies revealed that different policies are applied by biobanks in this respect [23, 39]. Considering the fact that it does not seem possible to formulate general criteria to decide on the return or destruction of leftover HBM, it seems undesirable to regulate it through formal legislation. However, biobanks could clarify in their access arrangements, which criteria the biobank will take into account to decide on the return or destruction of leftover HBM, such as the type, quality, reusability and amount of leftover HBM.

A significant number of informants pointed out that custodians and applicants tend to agree on the return or destruction of leftover HBM at the time of the approval of the access request. This implies that the custodian would (have to) negotiate with the applicant on a case-by-case basis on the return or destruction of leftover HBM. The ‘International Charter of principles for sharing bio-specimens and data,’ however, suggests that “control of the bio-specimens remains with Provider, who can at any time demand the return or destruction of data and bio-specimens if a breach in the agreement occurs” [34]. The final decision whether to return or destroy leftover HBM would thus remain with the custodian of the biobank.

Several informants are in favour of a trend to share HBM only between biobanks and not to provide any HBM directly to individual researchers. The exclusive exchange between biobanks would increase chances that the HBM and data are stored in a proper way. However, one should take into account that some individual researchers are not affiliated to an institution that hosts a biobank.

Some informants pointed out that it would be very burdensome and difficult – or even impossible – to verify whether the applicant had returned or destroyed all leftover HBM. That is why it was argued that return (or destruction) should be the exception rather than the rule. Instead, biobanks should – to the extent possible – divide HBM in different aliquots and provide only the minimum amount of HBM statistically relevant to obtain a successful outcome. Additional amounts of HBM could be provided in the course of the project.

### The right of biobanks to share in the benefits of a research project

Several informants suggested that it would be desirable that some of the benefits of using HBM and data in research projects are returned to the biobank infrastructure [10, 40]. A custodian of a biobank in Scandinavia stated the following in this respect:

“*I think it’s highly justifiable that some of the financial benefit that comes out of research based on biobanking should be fed back into the infrastructure, (…).”*

*“I need to get money from central sources, and that’s where I would want some kind of public sector, health-economic analysis and mechanism to feed some of the benefit back in. Because if we do it right, there are big winnings for healthcare authorities.*”

All informants seemed to agree that only stakeholders that provide a scientific contribution might be entitled to share in the benefits of a research project. Possible scientific contributions to a research project mentioned during the interviews were (a) an inventive step; (b) participation or advice in the development of the research hypothesis, method or execution of the research project; (c) advice on the optimal selection and use of HBM and data to respond to a particular research question; (d) the initial idea or initiative to collect a particular type of HBM and/or data; (e) an extensive characterisation of HBM; (f) the development of modified HBM (such as cell lines, plasmids); and/or (h) the preparation and cleaning up of data sets.

Guidelines of the International Committee of Medical Journal Editors and the Committee on Publications Ethics only grant authorship to individuals that participated in “*drafting the article or revising it critically for important intellectual content*” and “*the final approval of the version to be published*” [41]. The provision of HBM or data for a research project is not considered a sufficient ground to grant (co-) authorship*.* In some cases biobanks may provide important contributions to research projects via the collection, processing and organization of unique collections of HBM and data or the provision of scientific advice or assistance to the project. Arguably, the biobank should be recognized for such contributions [35, 42, 43].

A custodian of a biobank in Scandinavia stated the following in relation to the importance of biobanks for biomedical research:

“*Make no mistake, that impact of the HPV vaccine would not have happened at that speed without biobanks.*”

Most informants were sceptical about the idea that the biobank or the collectors of HBM and data should hold intellectual property rights (IPRs) on research results. Nor did they think that the biobank or the collectors of HBM and data should receive royalties in relation to the exploitation of such IPRs. A custodian from a biobank in Scandinavia – with previous experience in the pharmaceutical industry – stated the following in this respect:

“*For me, with my background, it’s hard to see how a royalty mechanism would work. Even though biobanks have compressed the time taken to getting the value, it’s still a long development time to get a drug to the market.*”

The interviews confirmed that the access fees charged by biobanks are often not sufficient to recover all costs in relation to the collection and storage of HBM and data. The informants agreed that different access/users fees for public and private or internal and external applicants could be justified by the fact that biobanks are mostly funded with public investments or by the research institution affiliated to a biobank. Both (academic) custodians and (industrial) applicants indicated that an industrial applicant might prefer paying a higher, but all-inclusive access fee at the beginning of the project. A number of authors support the idea of charging higher access fees to external or industrial applicants that did not contribute (directly or indirectly) to the collection of HBM and data [4, 38].

Informants in Sweden, Norway and the Netherlands suggested that applicants that intend to commercialize the results of a research project would pay a so-called ‘public contribution fee’, i.e., an additional fee or tax for the use of publicly funded collections of HBM and data. In return applicants would obtain the right to (commercially) exploit the potential benefits of their research project. Such a fee would not aim to generate profits, but to provide a (fair) return for the contribution of common goods or infrastructure to the commercialisation of research projects. One could refer in this respect to public contribution fees or tax charged for the exploitation of natural resources, such as gas or oil fields. Public contribution fees might help to avoid discussions about how important the contribution of a particular collection of HBM and data was for the final outcome of the project. The generated fees or taxes could be dedicated to the funding of public healthcare and research infrastructure, including biobanks. The HUGO Ethics Committee recommended in its Statement on Benefit Sharing that “*profit-making entities dedicate a percentage (e.g., 1–3 %) of their annual net profit to healthcare infrastructure and/or to humanitarian efforts.*” J. Bovenberg argued in favour of a specific tax on tissue and cell products directly developed from HBM and data “*as an effective, if indirect, mechanism for letting a community share in the benefits resulting from the efforts of the taxpayer concerned and to make a licensee pay for the exclusive use of natural resources*” [40]. The author did, however, argue that such a tax would only be due when a research project resulted in actual profit for the applicant.

Finally, some informants referred to the Bioresource Research Impact Factor (BRIF) initiative that aims to “*promote the sharing of bioresources by creating a link between their initiators or implementers and the impact of the scientific research using them*” [35, 44]. One could mention that biobanks already publish information on the research they support and list publications citing research results.

### The right of biobanks to share in the benefits of a research project

The informants agreed that applicants could be requested to share their research results when they use HBM and data from publicly funded biobanks (see Table 3). Some informants doubted whether the custodian should impose this return of research results as a condition to allow access to the collection, since this could discourage researchers from using the collection [45].

**Table 3:** Pre-conditions to share research results

Infrastructure to store and share research results	Consensus
Rules on access to research results	Consensus
Recognition of researcher that generated results	Consensus
Respect interest of researcher that generated results	Consensus

Previous studies have confirmed that an increasing number of biobanks [46] and funding bodies – such as NIH, the Wellcome Trust and the Dutch cancer association “KWF Kankerbestrijding” [47] – require researchers to make their research results publicly available. This requirement is motivated by the desire to maximize the use of results of publicly funded research [43, 48]. This would certainly be the case for HBM and data that are quite rare or require a lot of effort or investment to collect and analyse, such as whole genome sequencing data. Combining research results from different projects may speed up future research and avoid certain research to be conducted several times. Access to research results from previous projects could also allow researchers from multiple disciplines and with different experience to reinterpret and question the results. Furthermore, it would make it possible to study the generated data from new entry points or perspectives while working on new research questions. This is particularly important since biomedical research increasingly depends on multidisciplinary approaches and no researcher or research institute can avail of all possible expertise. Finally, one can refer to the fact that an increasing number of scientific journals require researchers to deposit the raw data of publications in public databases in order to verify the quality of research and to avoid fraud.

Another reason for a biobank to require applicants to share their research results is the possibility to enrich the collection of HBM and data. The biobank has an interest in obtaining additional information in relation to the collection in order to extend the characterization and understanding of HBM and data. Information from research projects could also be used in the future as a basis for the selection and collection of new data from specific subgroups of donors.

Some authors invoke the principle of reciprocity to justify the obligation to return research results: researchers could be expected to share their results with stakeholders that have made substantial contributions to the collection of HBM and data, such as biobanks, researchers collecting HBM and data and donors [35, 45]. One of the first documents to introduce such a requirement in the field of genomic research was the data release policy of the Human Genome project (HGP), known as the ‘Bermuda Principles’ (1996) [48]. Afterwards, several other international documents promoted sharing research results with other researchers and the community at large [35], such as the Fort Lauderdale Agreement (2003) [49], the OECD Principles and Guidelines for Access to Research Data from Public Funding (2007) [26], the Toronto Statement on Pre-publication Data Sharing (2009) and the Global Alliance for Genomics and Health’s White Paper (2013) [49].

The majority of the informants agreed that the publication of the results of research involving HBM and/or data is in itself insufficient to comply with the obligation to return or share the research. After all, often only a limited selection of the research results is actually included in publications. Several informants were in favour of returning and/or sharing also negative results of research projects.

Informants indicated that the return of research results is only useful if a number of conditions are fulfilled. First, the results must have been managed in a proper way by the researcher who generated them and sufficient quality control must have been conducted. Without such guarantee, it could be dangerous and misleading to use results from previous research projects. Furthermore, infrastructure needs to be available to store the research results in a proper way and to allow other researchers to access and use them [50]. Finally, clear rules should be defined to decide upon requests for access to such research results. Some informants suggested that different levels of access could be provided to different stakeholders, such as biobanks, funding agencies and future researchers.

Another suggestion brought forward by several informants was to involve the researcher who generated the research results in new research projects. After all, this researcher formulated the initial research hypothesis and developed the research protocol and could thus provide valuable information on how the research results were obtained and how they can be interpreted and used in further research. As some informants pointed out, it could be difficult to correctly interpret research results without the necessary background and knowledge on how the research results were generated. A custodian in Scandinavia made the following remark in this respect:

“*Raw data can be so vast and enormous, and unless you understand how to interpret it, itcan be just meaningless*”

The majority of the informants did express the opinion that the final decision to grant access to research results should not be taken by the initial researcher, but rather by the access committee of the biobank or a REC. This was motivated amongst other reasons by the possibility that the initial researcher may have a conflict of interest when deciding whether a new research project – possibly in the same or similar research domain – should be allowed to use his research results.

Researchers may be hesitant to share their research results if they do not receive recognition for their investments in generating the results [34]. Several authors have pointed out that the legitimate interests of the researchers and the institutions and funders supporting the project should be respected. Those interests may be the right to keep some research results confidential, to obtain IPRs in relation to research results and a priority right to publish research results [34, 35, 45, 48].

A final point raised by some informants was the question whether it is desirable or optimal for individual biobanks to store all research results generated with their collection of HBM and data. Some suggest that only biobanks or biobank networks with the necessary scientific expertise and knowledge may be suited to store and provide access to research results in a meaningful way. Others suggested that the research results would be stored in central research facilities, such as the European Genome-Phenome Archive (EGA).

## Conclusions

The interviews with different stakeholders revealed a rather complex web of rights and obligations allocated to the custodian and the applicant in relation to access to HBM and data stored in biobanks and used for research projects. The results did not allow creating a complete overview of the rights and obligations that the custodian and the applicant hold or should hold. Some rights and obligations are negotiated on a case-by-case basis, while others are stipulated in access arrangements. Furthermore, custodians and applicants can only exercise certain rights when they fulfil particular obligations and conditions.

There did seem to be a consensus on the attribution of certain general rights to the custodians and the applicant (see Table 4). First, the informants agreed that the custodian of a biobank should be able, under certain conditions, to decide upon access requests. However, there was no consensus on how extensive the evaluation of an access request should be and the extent to which the custodian of a biobank should decide upon the scientific merits of an access request. Second, nearly all informants agreed that a new request or an amendment to the initial request has to be submitted when an applicant wants to use leftover HBM in a new or follow-up project. Third, the informants agreed that different access fees might be applied to industrial or external applicants. Fourth, most informants agreed that a custodian of a biobank should be able to request the sharing and return of research results and that some of the benefits of research projects should be fed back into biobanks. There was no consensus on how the custodian would exercise such rights in practice and which conditions they would have to fulfil in this respect.

**Table 4:** Overview of main rights and obligations of custodians and applicants

1. Rights and obligations in relation to the decision on access to HBM and data	
A. Rights of custodian	Decide on access to HBM and data
	Evaluate availability and suitability of HBM and data for project
	Evaluate impact on existing collection of HBM and data
	Determine priorities between different research projects
B. Obligations of custodian	Have the disposal of sufficient expertise/experience
	Act independently
C. Rights of applicant	Access collection of HBM and data
	Use HBM and data in research projects
	Keep certain information confidential
	Priority access in case applicant collected HBM and data
D. Obligations of applicant	Use for specific project within certain time period
	Provide synopsis of project
2. Right to decide on leftover HBM	
A. Rights of custodian	Decide on return or destruction
	Decide on use in new or follow-up project
	Verify leftover HBM
	Provide minimum amount of HBM
B. Obligation of applicant	Inform biobank about leftover HBM
	Return or destroy leftover HBM
	Submit new request for new use of HBM
	Prohibition to transfer to third party
3. Right to participate in benefits of research project	
A. Rights of custodian	Charge access fee
	Request return of some of the benefits
	Share in benefits of research project
B. Right of applicant	Enjoy benefits of research project
C. Obligations of applicant	Pay access fee
	Return some of the benefits to biobank

We only included the rights and obligations on which most stakeholders agreed

## Additional files

Additional file 1: Interview guide. (PDF 57 kb) Additional file 2: Common list of concepts. (PDF 46 kb)
