# Supplementary material for: Qualitative study on custodianship of human biological material and data stored in biobanks
Source: BMC Med Ethics. 2016 Mar 1;17:15. doi: 10.1186/s12910-016-0098-0 (PMC4772467; doi:10.1186/s12910-016-0098-0)
Supplement: Additional file 1: — Interview guide. (PDF 57 kb) [file 12910_2016_98_MOESM1_ESM.pdf]

Annex 1: Interview guide for semi-structured interviews

1. Factual biography
   1.1. Interview date
   1.2. Name of informant
   1.3. Position of informant
   1.4. Short description of the relationship of the informant with biobanking

2. Short description of purpose and nature of the interview

   - The current research would like to study practical experience and personal opinion on hopes and concerns regarding the relationship between custodians and researchers/PI

3. Guidelines

   - The informant's identity remains anonymous in the written report of the study and responses are treated in confidence. There will only be references to the concerned biobank or function, but not the combination of both, since this would allow identification of the informant
   - Request permission to tape record interview

4. Checklist of topics
   4.1. Questions in relation to access to biobanks:
      i. What is your professional experience with biobanking?
         a. How is your institute involved in biobanking?
      ii. How to handle requests for access?
         a. What is the policy at your institute? What are the rules in your country?
         b. What are your experience and your own opinion in this respect?
         c. Is the access policy/arrangement of your institute publicly available?
      iii. Does a different policy apply to access to HBM and access to data?
      iv. What should happen with leftover HBM?
         a. What is the policy at your institute? What are the rules in your country?
         b. What are your experience and your own opinion in this respect?

v. Which benefits can result from use of HBM and data in research projects?

- Prompts: To which extent could a biobank participate in such benefits? When?
    - What is the policy at your institute? What are the rules in your country?
    - What are your experience and your own opinion in this respect?

vi. What is understood by returning 'research results'

- Is 'Returning research results' a category of benefits?
    - What is the policy at your institute? What are the rules in your country?
    - What are your experience and your own opinion in this respect?
    - Should researcher that generated research results be involved in decision on use of returned results?
        - What if HBM was prospectively collected at the request of the applicant?

vii. Publicly available information on access to HBM and/or associated data?

- Prompts: How could one increase transparency?
    - What is the policy at your institute? What are the rules in your country?
    - What are your experience and your own opinion in this respect?
- Would you be in favour of a registration obligation as exists for clinical trials?

viii. Access by industry:

- Should industry be allowed access to public biobanks?
- Should a different policy apply to industrial applicants?

4.2. Open question: Are there any other hopes or concerns in relation to access to biobanks?

5. Closing comments
    5.1. Debriefing
    5.2. Copy of final report will be sent
