# Supplementary material for: Qualitative study on custodianship of human biological material and data stored in biobanks
Source: BMC Med Ethics. 2016 Mar 1;17:15. doi: 10.1186/s12910-016-0098-0 (PMC4772467; doi:10.1186/s12910-016-0098-0)
Supplement: Additional file 2: — Common list of concepts. (PDF 46 kb) [file 12910_2016_98_MOESM2_ESM.pdf]

Aggregated dimensions	2nd order themes	1st order concepts
1. Strategy	Partnership/collaboration	
2. Access requests	2.1 Decision body	Funding body
		Sample access committee
	2.2 Decision criteria	HBM from rare diseases or rare HBM
		Scientific value/merit: Research questions
		Quality of research (study design, scientific validity, statistical relevance)
		Societal or medical relevance/benefit
		Commercial or not
		Approval by ethics committee or other body
		Research experience/merit
		Other criteria
		Correspondence with vision
	2.3 Extent of detail	Abstract or complete protocol
		Amount and type of HBM and type of patients required
		Research hypothesis/objective
		Statistical relevance of sample size
		Other
		Research method/technique
3. Return and/or destruction of left-over HBM	3.1 Criteria for return	Prior agreement
		Rare vs. common HBM
		Other
		Quality/reusability of returned HBM
	3.2 Criteria for destruction	Complexity
		Cost
	3.3 Criteria for re-use	Amendment/Full review
	3.4 Alternatives	Providing limited number/sharing in parts
4. Participation in benefits	4.1 Criteria for benefit sharing	(scientific)contribution/inventive step
		Other
		Collaboration (agreement)
	4.2 Mechanism of benefit sharing	Sharing of IP/royalty/upfront payment
		Public contribution fee/central funding
		Other
		User fee/cost recovery
5. Return, sharing and publication of research results	5.1 Advantage of return	Avoid duplication and unnecessary costs
		Enrich collection
		Share scientific knowledge
		Quality control
		Reinterprete data with new expertise
	5.2 Criteria for return	Quality and reusability of results
		Infrastructure/storage capacity
		Respect interest researcher
		Other
		Protection of personal integrity
	5.3 Criteria for not return	Difficulty to interprete
		Competitive advantage
	5.4 Obligatory or not	Requirement for access (to additional data)
	5.5 Type of data/HBM	Negative results/validated results/raw data
		Modified HBM
	5.6 Power to decide	Suggestion/obligation to collaborate
		Decision to share reseach results

**Aggregated dimensions**	**2nd order themes**	**1st order concepts**
**Aggregated dimensions**	**2nd order themes**	**1st order concepts**
6. Initiative for collection	6.1 Initiative biobank	Use for research specified in request
		Single 'delivery' fee
		More rights for biobank
	6.2. Initiative collector	(transfer of) Decision (right) to use HBM for project
		Fee for services/entire concept
		Priority right to use/publish
7. Exclusive access	7.1 Why not?	
	7.2 Alternative concepts	Priority right/preferential access
8. Publicly available information	8.1 Which information	Line of research
		Commercial non sensitive information
		Abstract
	8.2 Advantage	Avoid duplication; Enable synergies/collaborations; Knowledge about existing research/failures
